# RETRACTED: Jarero-Basulto et al. Cytotoxic Effect of Amyloid-β1-42 Oligomers on Endoplasmic Reticulum and Golgi Apparatus Arrangement in SH-SY5Y Neuroblastoma Cells. *NeuroSci* 2024, *5*, 141–157

**DOI:** 10.3390/neurosci6040127

**Published:** 2025-12-10

**Authors:** José J. Jarero-Basulto, Yadira Gasca-Martínez, Martha C. Rivera-Cervantes, Deisy Gasca-Martínez, Nidia Jannette Carrillo-González, Carlos Beas-Zárate, Graciela Gudiño-Cabrera

**Affiliations:** 1 Cellular Neurobiology Laboratory, Cell and Molecular Biology Department, University Center of Biological and Agricultural Sciences (CUCBA), University of Guadalajara, Zapopan 45220, Mexico; jose.jarero@academicos.udg.mx (J.J.J.-B.); mrivera@academicos.udg.mx (M.C.R.-C.); 2 Development and Neural Regeneration Laboratory, Cell and Molecular Biology Department, University Center of Biological and Agricultural Sciences (CUCBA), University of Guadalajara, Zapopan 45220, Mexico; yadira.gasca3953@alumnos.udg.mx (Y.G.-M.); nidia.carrillo@academicos.udg.mx (N.J.C.-G.); 3 Behavioral Analysis Unit, Neurobiology Institute, Campus UNAM, Juriquilla 76230, Mexico; gasca@inb.unam.mx; 4 Neurobiotechnology Laboratory, Cell and Molecular Biology Department, University Center of Biological and Agricultural Sciences (CUCBA), University of Guadalajara, Zapopan 45220, Mexico; carlosbeas55@gmail.com

The journal retracts the article “Cytotoxic Effect of Amyloid-β1-42 Oligomers on Endoplasmic Reticulum and Golgi Apparatus Arrangement in SH-SY5Y Neuroblastoma Cells” [1] cited above.

Following publication, the authors contacted the Editorial Office to raise concerns regarding a potential lack of permission to publish the data contained within this article [1].

Adhering to our standard procedure, an investigation was conducted by the Editorial Office and Editorial Board that confirmed that appropriate permission to publish the data had not been gained and that retrospective approval could not be obtained. Consequently, the Editorial Board and the authors have decided to retract this publication [1], as per MDPI’s retraction policy (https://www.mdpi.com/ethics#_bookmark30).

This retraction was approved by the Editor-in-Chief of the journal *NeuroSci*.

The authors agreed to this retraction.

## References

[1] Jarero-Basulto J.J., Gasca-Martínez Y., Rivera-Cervantes M.C., Gasca-Martínez D., Carrillo-González N.J., Beas-Zárate C., Gudiño-Cabrera G. (2024). RETRACTED: Cytotoxic Effect of Amyloid-β1-42 Oligomers on Endoplasmic Reticulum and Golgi Apparatus Arrangement in SH-SY5Y Neuroblastoma Cells. NeuroSci.

